# Substituting Chemical by Organic Fertilizer Improves Soil Quality, Regulates the Soil Microbiota and Increases Yields in *Camellia oleifera*

**DOI:** 10.3390/microorganisms13112509

**Published:** 2025-10-31

**Authors:** Li Wen, Hanfang Luo, Chao Li, Kaikai Cheng, Lihong Shi, Lingling Liu, Ke Wang, Haiming Tang

**Affiliations:** 1 Hunan Cultivated Land and Agricultural Eco-Environment Institute, Changsha 410125, China; wenli@hunaas.cn (L.W.);; 2 College of Resource, Hunan Agricultural University, Changsha 410128, China

**Keywords:** organic fertilizer, soil microbial community, yield enhancement, soil bacteria, metagenomic sequencing

## Abstract

The partial substitution of chemical fertilizer with organic fertilizer has been regarded as an effective strategy for enhancing crop yield and soil quality. Nevertheless, its effects on soil properties and microbes remain contentious. In this study, we examined the effects of four different fertilization strategies (including without fertilizer (CK), 100% chemical fertilizer (NPK), 30% organic fertilizer + 70% chemical fertilizer (LOM) and 60% organic fertilizer + 40% chemical fertilizer (HOM)) on soil nutrients and microbial communities through metagenomic sequencing in a *Camellia oleifera* field experiment. Compared to CK and NPK, HOM significantly increased SOC, TN, TP, AK and AN contents. The substitution of organic fertilizer notably increased *Camellia oleifera* yield, with the highest increase of 93.35% observed in HOM relative to NPK. Soil bacterial and fungal communities responded inconsistently to fertilization patterns. Bacteria predominated as the main soil microorganisms, and higher rates of organic fertilizer substitution facilitated a shift from bacterial to fungal communities. Organic fertilizer substitution significantly increased soil bacteria diversity and fungal richness, particularly in the HOM. Soil bacterial community structure was more sensitive to fertilization regimes than soil fungi. High rates of organic fertilizer substitution substantially suppressed oligotrophic and increased copiotrophic bacterial communities. *Mucoromycota* emerged as the dominant fungal group, with a considerable increment in HOM soils. SOC and TN were the main factors affecting *Camellia oleifera* yield and shaping soil bacteria and fungal diversity and composition. This study provided crucial insights into the ecological implications of organic fertilizer application and the potential of managing soil microorganisms for sustainable *Camellia oleifera* productivity.

## 1. Introduction

*Camellia oleifera* (*C. oleifera* hereafter) is an economically significant forest tree endemic in China [1]. Monounsaturated fatty acids, polyphenols, vitamin E and other active ingredients are prevalent in *C. oleifera* oil. These constituents confer multiple health benefits, including regulating lipid metabolism, enhancing immune function, and preventing cardiovascular diseases [1]. Traditionally, fertilization practices for *C. oleifera* have been limited to winter applications or omitted altogether due to the crop’s relatively low economic returns, thereby constraining the sustainable development of the *C. oleifera* production [2]. In response to increasing market demand for *C. oleifera* oil production, excessive chemical fertilization has been widely conducted in many plantations. However, intensive chemical fertilization has been attributed to environment challenges like water eutrophication and elevated emission of greenhouse gases, as well as aggravating major soil degradation problems consisting soil acidification, soil nutrient depletion and microbial biomass and diversity decline [3,4]. These challenges underscore the urgent need to develop sustainable soil fertility management strategies that can improve *C. oleifera* productivity while mitigating adverse environment impacts.

Recently, organic fertilizers have been developed and extensively utilized as partial substitutes for chemical fertilizers in agricultural production, aiming to improve soil quality and produce high-quality crops [5,6]. Organic fertilizers are capable of mitigating difficulties connected with synthetic fertilizers by reducing the need for frequent applications of chemical inputs to maintain soil fertility by gradually releasing nutrients into the soil solution and maintaining nutrient balance for healthy crop growth. Additionally, they serve as a valuable energy source for soil microbes, which contributes to improve soil structure and promotes crop growth [7,8,9]. Nonetheless, the nutrients progressively supplied by substituting organic fertilizers usually fail to meet crop demands during peak growth stages [10]. Therefore, the combined application of organic and chemical fertilizers has been proposed to addresses this limitation by delivering both immediate nutrients availability and long-term soil conditioning, while also facilitating nutrient balance [10]. Empirical studies have demonstrated that partially substituting chemical fertilizers with organic fertilizers can improve soil properties and increase crop yield [10,11].

Soil microbes play a crucial role in plant growth and health, as well as many vital soil ecosystem processes such as soil nutrient cycling [12]. They facilitate organic matter decomposition and regulate the nutrient availability for plants, rendering them vital for maintaining soil fertility [12]. The composition, diversity and functional capacities of soil microbes serve as sensitive indicators of soil health and ecosystem productivity [13]. Chemical fertilization can lead to considerable decline in soil microbial diversity and abundance, which is typically associated with reduced soil quality, particularly at high rates of chemical fertilizer application [14,15]. In contrast, organic fertilizers supply organic matter as an energy source for microbial growth. Appropriate application of organic fertilizers can enhance soil physicochemical properties, modulate the structure of soil microbial communities, improve soil fertility, and consequently promote crop growth [16,17]. For instance, Bebber D P and Richards V R [17] reported that organic fertilization substitution had a more pronounced effect on soil bacterial diversity compared to both chemical and non-fertilization, although no discernible variations in fungal diversity were observed between fertilized and unfertilized soil. However, when organic fertilization completely and solely replaces chemical fertilizer, changes in soil microbial diversity are minimal. Furthermore, the structure and composition of soil microbial communities, along with their functional groups, vary according to different fertilization practices [11,18]. These inconsistent findings suggest that further research is necessary to evaluate the fluctuations in soil microbial communities resulting from the application of varying proportions of organic fertilizer.

In this study, we hypothesized that partial substitution of chemical fertilizer with organic fertilizer would improve soil microbial diversity and the yield of *C. oleifera.* To test this hypothesis, we conducted a field experiment to (1) evaluate the effects of different fertilization strategies on *C. oleifera* yield, soil physicochemical properties, and soil microbial diversity and community composition; and (2) determine the relationships among plants yield, soil properties and soil microbial communities under different fertilization regimes. The results will provide theoretical guidance for optimizing fertilization management approaches to ensure the sustainable utilization of the *C. oleifera* industry.

## 2. Materials and Methods

### 2.1. Site Description

This study was conducted in Changning City, Hunan Province, China (26°16′48″ N, 112°12′36″ E). The region experiences a humid subtropical monsoon climate, characterized by a mean annual temperature of 18.1 °C and annual precipitation of 1436 mm. The rainy season usually spans from April to June. According to the Chinese soil classification, the soils in this area primarily develop on acidic purple sandstone. At the beginning of this field experiment, the soil chemical characteristics of the 0–10 cm soil layer were as follows: pH: 4.76; soil organic carbon (SOC): 2.95 g kg^−1^; total nitrogen (TN): 0.30 g kg^−1^; total phosphorus (TP): 0.09 g kg^−1^; total potassium (TK): 17.38 g kg^−1^; available phosphorus (AP): 0.63 mg kg^−1^; available potassium (AK): 59.80 mg kg^−1^; and available nitrogen (AN): 45.60 mg kg^−1^.

A randomized complete block design field experiment was established in April 2023. Each block included four fertilization treatments: no fertilizer (CK, control), only chemical fertilizer (NPK), 30% organic fertilizer mixed with 70% chemical fertilizer (LOM), and 60% organic fertilizer mixed with 40% chemical fertilizer (HOM). Each treatment set has four replications. The area of each test block was 270 square meters (3.0 m width × 90.0 m length). The experimental field was consistently planted with *C. oleifera* for four years, adhering to local agronomic management practices. The fertilizers utilized were “Xiyang” compound fertilizer (N-P_2_O_5_-K_2_O: 15-15-15) and “Wangsai” compound organic fertilizer (organic matter > 20%, N-P_2_O_5_-K_2_O: 15-6-4, CFU > 20 million g^−1^).

### 2.2. Sampling Collection and Measurement

#### 2.2.1. Soil Sampling and Methods of Soil Properties

Soil sampling (0–20 cm soil layer) was conducted in October 2023, coinciding with the oil tea harvesting period. Five points were randomly selected within each replicate and then collected and mixed as one sample. In addition, two 60 × 60 × 30 cm^3^ soil pits were excavated and collected using metal rings to measure soil bulk density (BD). After removing stones and roots, a portion of the fresh soil samples was freeze-dried and promptly stored at −80 °C for metagenomic analysis. The remaining portion of the soil samples were air-dried and passed through a 0.15 mm sieve for the measurement of the soil’s physical and chemical properties.

Soil water content (SWC) was determined by drying the soil at 105 °C for 48 h and weighted. Soil pH was measured using a pH meter (FE20K, Mettler Toledo, Greifensee, Switzerland) at a soil/water ratio of 1:2.5. The content of SOC and TN were determined by K_2_CrO_7_-H_2_SO_4_ digestion and semi-micro Kjeldahl digestion, respectively. Soil TP was quantified using the molybdenum blue colorimetric method. Soil AN was detected via the alkaline permanganate method, while soil AP was measured using the molybdenum blue colorimetric method. Soil AK was analyzed by inductively coupled laser atomic emission spectroscopy (ICP-AES). Additionally, methods for detecting soil properties have been documented previously [19].

#### 2.2.2. Plant Yield Determination

At the harvesting time on 23 October 2023, five representative *C. oleifera* plants were randomly selected from each plot to collect all *C. oleifera* fruits, and all fruits yields were weighted and calculated. We used the coefficient of variation (CV) and contribution rate of fertilizer (FCR) as two metrics to compare the yield stability and yield-increasing effect of organic fertilizer substitution [20,21]. The greater the CV, the more volatile the crop yield under specific management practices [21]. The calculations for CV and FCR were performed using the following equation:

(1) $$\mathrm{CV}(\%)\;=\frac{\sigma }{\gamma }$$

(2) $$\mathrm{FCR}(\%)=\frac{\gamma F-\gamma \mathrm{NPK}}{\gamma F}\;\times \;100$$

where σ is the standard error (kg hm^−2^), γ is the average yield (kg hm^−2^), and γ_F_ and γ_NPK_ present the average yield of organic fertilizer substitution and only chemical fertilization practice, respectively.

#### 2.2.3. Genomic DNA Extraction and Metagenomic Sequencing

The genomic DNA of the soil was extracted using a DNA extraction kit, and its concentration was determined by agarose gel electrophoresis and NanoDrop 2000 (Thermo Fisher Scientific, Waltham, MA, USA). A 130 μL (260 ng) DNA sample was transferred to a Covaris tube (Covaris, Woburn, MA, USA) for fragmentation. Subsequently, 100 ng of fragmented DNA was subjected to end-repair and adapter-ligation steps, followed by magnetic bead purification and size selection, library amplification, and product purification using Quantify library concentration with Qubit3.0. We used Agilent 2100 (Agilent, Santa Clara, CA, USA) to check the length of the library.

The Metagenome sequencing and subsequent analysis were performed by OE Biotech Co., Ltd. (Shanghai, China). The raw data was obtained in FASTQ format. Reads were trimmed and filtered using fastp (version 0.20.1) [22]. Host pollution control was needed if the DNA was extracted from host-associated environments. The post-filtered pair-end reads were aligned against the host genome using bbmap (version 38.93-0) and the aligned reads were discarded. Valid reads were then subjected to metagenomic assembly using MEGAHIT (version 1.2.9) [23]. The open reading frames (ORFs) were predicted from assembled contigs (longer than 500 nt) using Prodigal (version 2.6.3), and these ORFs were translated into amino acid sequences [24]. To generate a non-redundant gene catalog, all predicted genes were clustered using MMSeqs2 (version 13.45111) with clustering criteria set at 95% sequence identity and 90% coverage [25]. The longest gene within each cluster was selected as the representative sequence. Clean reads from each sample were aligned against the non-redundant gene set at 95% identity using Salmon (version 1.8.0), enabling quantification of gene abundance per sample. The gene set representative sequence (amino acid sequence) was annotated with NR database with an e-value of 1 × 10^−5^ using DIAMOND (version 0.9.10.111) [26]. Species annotations were obtained from the corresponding taxonomic information database of the NR library, and the abundance of the species was calculated by summing up the corresponding gene abundance of the species and counting the species at each taxonomic level of kingdom, phylum, class, order, family, genus, and species.

### 2.3. Statistical Analysis

Prior to analysis, the data were assessed for normality and homogeneity, and natural logarithmic transformations were performed where necessary. One-way analysis of variance (ANOVA) was conducted to examine significant differences in soil properties and soil microbial diversity across all fertilization treatments. Principal coordinates analysis (PCoA) based on Bray–Curtis distances was applied to reveal changes in soil microbial community structure. The influences of fertilization on soil fungal and bacterial community structures were examined by permutational multivariate analysis of variance (PERMANOVA). All statistical analyses were performed using SPSS 21.0 (SPSS, Inc., Chicago, IL, USA). Differences were considered significant if the *p* value was less than 0.05.

For the relationships between soil physicochemical properties and soil microbial diversity and structure were assessed using redundancy analysis (RDA). Stepwise multiple linear regression was used to identify and quantify the contributions of the most influential explanatory variables affecting soil microbial communities. Linear discriminant analysis of effect size (LEfSe) was applied to discover biomarkers. We used the linear discriminant analysis (LDA) effect size (LEfSe) algorithm to identify taxa with differentiating abundance in the different treatments [27]. Then, LDA linear discriminant analysis (LDA > 3.0) was performed to estimate the relative contribution of these taxa to the observed differences among the four treatment groups.

## 3. Results

### 3.1. Soil Properties and Plant Yield

**Figure 1:**
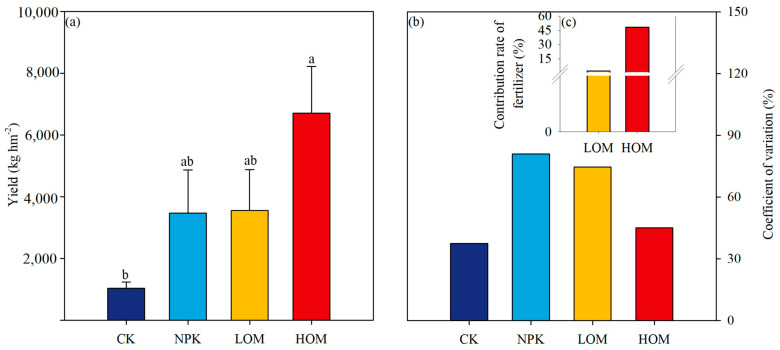
Indicators of *Camellia oleifera* yield (**a**), coefficient of variation (**b**) and contribution rate of fertilizer (**c**) in different fertilization treatments. Different lowercase letters above columns indicate significant differences among the treatments at *p* < 0.05. CK: without fertilizer, NPK: 100% chemical fertilizer, LOM: 30% organic fertilizer + 70% chemical fertilizer, HOM: 60% organic fertilizer + 40% chemical fertilizer.

Soil chemical properties were significantly affected by fertilization treatments (Table 1). Soil pH exhibited a notable decrease across all fertilization regimes (Table 1, *p* < 0.05). Compared to the CK treatment, soil pH in the NPK, LOM, and HOM treatments decreased by 0.45, 0.62, and 0.42 units, respectively (Table 1). In contrast, soil TN and SOC content displayed divergent responses. The highest soil TN value (0.58 ± 0.15 g kg^−1^) and SOC (5.09 ± 0.95 g kg^−1^) were observed in HOM treatment, surpassing other treatments by 4.7 to 7.8 times and 1.98 to 2.5 times, respectively (*p* < 0.05). Both LOM and HOM also significantly increased the content of soil TP (Table 1, *p* < 0.05). Fertilization effects varied across nutrient availability indices. For instance, soil AN significantly increased in HOM (264 ± 27.3 mg kg^−1^), showing an 801% increase relative to CK while NPK and LOM treatments showed minimal promotion compared to CK. In contrast, soil AP contents remained at a constant level across all fertilization treatments (*p* > 0.05). The stoichiometric ratios of carbon, nitrogen, and phosphorus exhibited insignificant shifts among the four treatments (Table 1, *p* > 0.05). In comparison to CK, NPK, LOM and HOM treatments resulted in a two-to-five-fold increase in the fruit yield of *C*. *oleifera*, with the highest yield observed in HOM treatments (Figure 1a). The CV in HOM was lower than that in NPK and LOM treatments (Figure 1b). The FCR in HOM was 20 times that in LOM (Figure 1c).

### 3.2. Soil Microbial Composition and Diversity

#### 3.2.1. Composition of Soil Bacteria and Fungi Communities

The results of the soil metagenome sequencing are shown in Table S1. The raw sequences generated from the metagenome sequencing were trimmed and filtered, and more than 1.2 billion sequenced sequences (reads) were obtained from all samples, which are suitable for operational classification and functional analysis. A total of 9,160,198 contigs were derived from microbial macrogenomes (Table S1). In addition, the coverage of all samples for soil bacteria and fungi exceeded 99.9%, suggesting that the sequencing depth in this study was sufficient for diversity analysis (Table 2).

**Figure 2:**
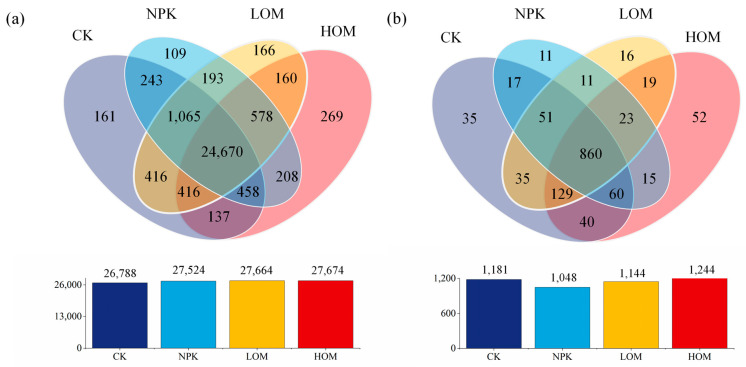
Venn diagram of the number of shared and unique species in the different fertilization treatments in bacteria (**a**) and fungi (**b**). Only the OTUs present in two biological replicates of each sample were retained. CK: without fertilizer, NPK: 100% chemical fertilizer, LOM: 30% organic fertilizer + 70% chemical fertilizer, HOM: 60% organic fertilizer + 40% chemical fertilizer.

Metagenomic sequencing analysis identified a total of 169 phyla, 4118 genera, and 29,249 species in bacteria, as well as 10 phyla, 714 genera, and 1374 species in fungus. For bacteria, Venn diagram analysis revealed that there were 26,788, 27,524, 27,664 and 27,674 species in CK, NPK, LOM, and HOM soils, respectively (Figure 2a). Among them, there were 161, 109, 166 and 269 unique bacterial species in CK, NPK, LOM, and HOM (Figure 2a). These unique species accounted for between 0.37% and 0.91% of all species in four treatments (Figure 2a). Furthermore, there were 24,670 common bacterial species, constituting approximately 84.3% of the total species pool (Figure 2a). With fungi, CK, NPK, LOM, and HOM soils contained 1181, 1048, 1144 and 1244 species, respectively (Figure 2b). Among them, four treatments soil contained 35, 11, 16, and 52 unique fungus species, respectively (Figure 2b). These unique species contributed between 0.8% to 3.78% of all species in four treatments (Figure 2b). Additionally, 860 fungal species were common to all four treatments, accounting for about 62.6% of the total fungal species identified (Figure 2b).

#### 3.2.2. Soil Bacterial and Fungal Community

The soil bacteria and fungi diversity exhibited differential responses to fertilization regimes (Table 2). For soil bacteria, both the LOM and HOM treatments significantly increased the Shannon and Simpson indices compared to CK (Table 2, *p* < 0.05). However, no statistically significant differences were observed in the Chao1 and ACE richness indices of bacteria across the four treatments (Table 2, *p* > 0.05). In contrast, the HOM treatment showed a significant increase in Chao1 and ACE indices of soil fungi and a notable reduction in the Shannon and Simpson indices (Table 2, *p* < 0.05). NPK significantly reduced the index of Chao1 and ACE (Table 2, *p* < 0.05). The fungi Shannon and Simpson indices in LOM were significantly higher than that in HOM (Table 2, *p* < 0.05).

**Figure 3:**
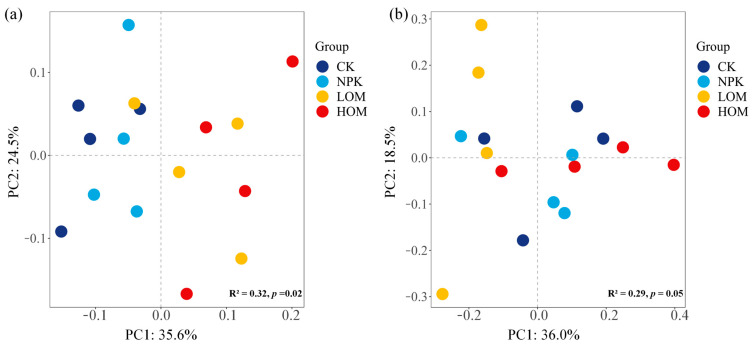
Bacteria (**a**) and fungi (**b**) betadiversity of *Camellia oleifera* plantations soil in different fertilization treatments based on the PCoA result. CK: without fertilizer, NPK: 100% chemical fertilizer, LOM: 30% organic fertilizer + 70% chemical fertilizer, HOM: 60% organic fertilizer + 40% chemical fertilizer.

PCoA analysis was used to evaluate the differences in the structure of bacterial and fungal communities across four fertilization treatments (Figure 3). Permutational multivariate analyses indicated that fertilization regime had a substantial influence on community compositions (*p* < 0.01). The first and second principal components explained approximately 60.1% and 54.5% of the variation in bacterial and fungal communities, respectively (Figure 3). The sample points in LOM and HOM soils were distinctly separated from those in CK and NPK soils regarding soil bacteria (Figure 3a). In contrast, the four treatments did not exhibit clear differentiation for soil fungi (Figure 3b).

#### 3.2.3. Soil Bacterial and Fungal Structure and Composition

**Figure 4:**
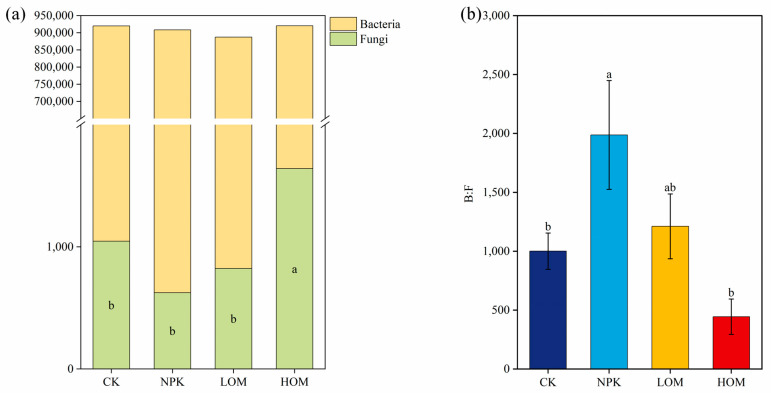
Total abundance of soil bacteria and fungi (**a**) and the ratio of soil fungi to bacteria abundance (**b**). Data present means ± standard error, different lowercase letters represent significant difference among four fertilization treatments (*p* < 0.05). CK: without fertilizer, NPK: 100% chemical fertilizer, LOM: 30% organic fertilizer + 70% chemical fertilizer, HOM: 60% organic fertilizer + 40% chemical fertilizer.

Among all treatments, soil bacteria was dominant with 95.5% to 98.5% total abundance at the species level, whereas fungal communities occupied only 0.05% to 0.17% across all soil samples (Figure 4a). Relative to CK, other three fertilization treatments tended to reduce the total abundance of soil bacteria. On the contrary, the abundance of fungi exhibited an increasing trend under the HOM treatment (Figure 4a). Further analysis revealed that the highest B: F ratio existed in NPK soil, while the lowest value was observed in HOM (Figure 4b).

**Figure 5:**
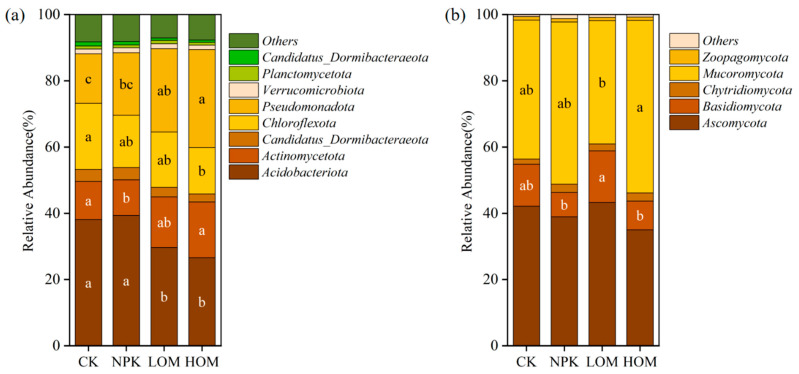
Community composition of soil bacteria (**a**) and fungi (**b**) under different fertilization treatments (phylum level, relative abundance > 1%). Different lowercase letters represent significant difference among four fertilization treatments (*p* < 0.05). CK: without fertilizer, NPK: 100% chemical fertilizer, LOM: 30% organic fertilizer + 70% chemical fertilizer, HOM: 60% organic fertilizer + 40% chemical fertilizer.

In this study, the dominant phylum of microorganisms with relative abundance greater than 1% was analyzed. Bacterial communities were dominated by *Acidobacteriota* (33.4%), *Pseudomonadota* (22.1%), *Actinomycetota* (13.6%), and *Chloroflexota* (16.6%), and LOM and HOM soil significantly reduced the relative abundance of *Acidobacteriota* and *Chloroflexota*, while *Pseudomonadota* abundance increased compared to that in CK (Figure 5a, *p* < 0.05). No significant differences were observed in the relative abundance of the abovementioned three phyla in CK and NPK soils (Figure 5a, *p* > 0.05). In addition, the relative abundance of oligotrophic communities was significantly higher in LOM and HOM soil, whereas copiotrophic communities were more abundant in CK and NPK soils (Figure S1, *p* < 0.05). Consequently, the ratio of copiotrophic to oligotrophic (C:O) was significantly higher in LOM and HOM soils than that in CK and NPK soils (Figure S1, *p* < 0.05). Further taxonomic classification indicated that the dominant bacterial taxa belonged to the *Acidobacteriota*, *Actinomycetota*, *Chloroflexota,* and *Pseudomonadota* phyla. These bacterial taxa exhibited a differential response to four fertilization treatments. Compared with CK, the HOM treatment significantly reduced the abundance of *Acidobacteriota*, *Acidobacteriaceae,* and *Chloroflexota*, while the abundance of *Gammaproteobacteria* increased significantly (Table S2).

In terms of fungi, *Mucoromycota* (53.4%) and *Ascomycota* (33.6%) were more prevalent in all soil fungal communities (Figure 5b). Compared with NPK, HOM significantly increased the relative abundance of *Mucoromycota* and decreased the relative abundance of *Basidiomycota* (Figure 5b, *p* < 0.05). However, no significant differences in the relative abundance of these two phyla were observed in CK and LOM soils (Figure 5b, *p* > 0.05). The dominant fungal species (relative abundance ≥ 1%) mainly belonged to the phyla *Mucoromycota*, *Basidiomycota,* and *Ascomycota* (Table S2). Compared with CK, LOM significantly decreased the abundance of *Rhizophagus irregularis*, *Rhizophagus clarus*, *Glomus cerebriform* and *Rhizophagus* sp. *MUCL 43196* (Table S2, *p* < 0.05). HOM significantly increased the abundance of *Rhizophagus_clarus, Glomus cerebriform*, *Rhizophagus_*sp.*_MUCL_43196* (Table S2, *p* < 0.05).

**Figure 6:**
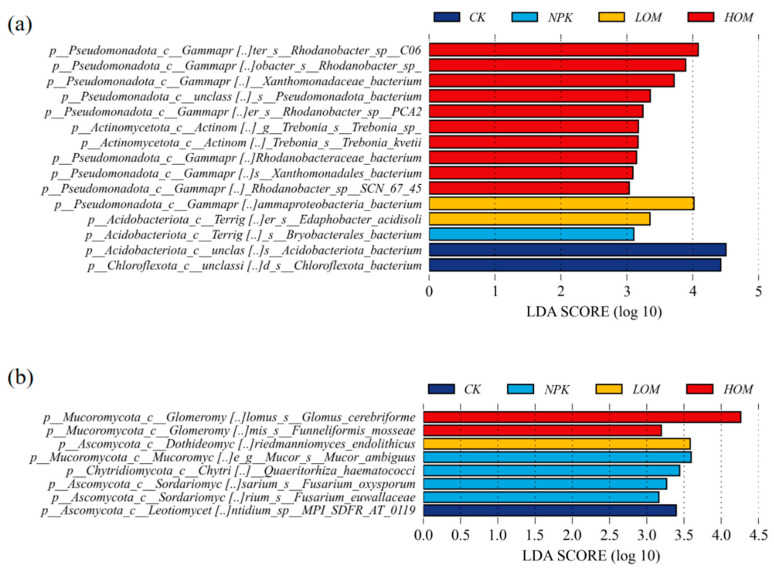
The biomarkers of soil bacteria (**a**) and fungi (**b**) of *Camellia oleifera* plantation soil. CK: without fertilizer, NPK: 100% chemical fertilizer, LOM: 30% organic fertilizer + 70% chemical fertilizer, HOM: 60% organic fertilizer + 40% chemical fertilizer.

In this study, soil biomarker taxa were systematically identified under different fertilization treatments utilizing the LEfSe method (Figure 6). Based on LDA scores (threshold LDA > 3.0) and statistical significance (*p* < 0.05), the characteristic species leading to differences between groups were screened. Among the bacterial taxa, there are 2, 1, 2 and 10 biomarkers in CK, NPK, LOM and HOM treatments, respectively (Figure 6a). In contrast, there are only 1, 4, 1 and 2 fungi biomarkers in CK, NPK, LOM and HOM treatments, respectively (Figure 6b). Notably, the biomarker taxa were entirely distinct across the four treatment soils. For bacteria, over half of the members of the bacterial biomarker group belonged to the phylum *Pseudomonadota*, and these bacterial biomarkers were mainly located in HOM treatment (Figure 6a). In terms of soil fungi, all identified signature fungal groups predominantly belonged to the phylum *Ascomycota* (Figure 6b). Specifically, the highest LDA score of the fungi was *Glomus cerebriforme* from *Mucoromycota* (Figure 6b).

### 3.3. Factors Potentially Influencing Soil Fungal and Bacterial Community and Plant Yield

**Figure 7:**
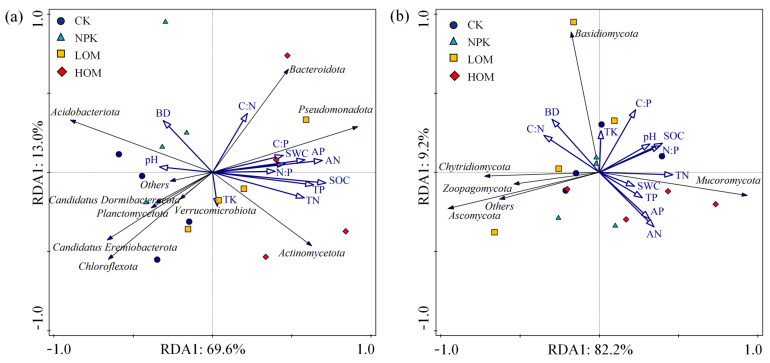
Redundancy analysis (RDA) of soil bacteria (**a**) and fungi (**b**) community structure constrained by soil chemical properties in different fertilization treatments.

Redundancy analysis (RDA) was used to analyze the relationship between soil fungal and bacterial communities and soil properties (Figure 7). The first two axes of RDA explained 82.6% and 91.4% of the variance in the bacterial and fungal communities, respectively, in relation to soil properties (Figure 7). Stepwise multiple linear regression analysis indicated that TN was the main explanatory variable for soil bacteria and fungi (Table S3). Spearman correlation analysis revealed a negative correlation between soil bacterial diversity and soil AN and AP, whereas soil fungal diversity was positively correlated with several soil properties including SWC, AN, AP, SOC, TN and TP (Figure S2, *p* < 0.01). The ratio of C:O was significantly positively correlated with soil nutrients including AN, AP, SOC, TN and TP (Figure S2, *p* < 0.01). The *C. oleifera* yield was notably positively correlated with soil properties (SWC, SOC, TN and AN), bacteria communities (*Actinomycota*, copiotophic taxa, C:O), and fungi diversity (Shannon index), and negatively correlated with *Acidobacteria*, oligotrophic taxa and BD (Figure S2, *p* < 0.01).

## 4. Discussion

### 4.1. Effects of Different Fertilization on Soil Properties and Yield

Fertilization, particularly organic fertilization, has received extensive attention owing to its vital role in proving nutrient utilization, enriching soil fertility and increasing crop yield [28]. In our study, chemical fertilizer reduction combined with organic fertilizer has a positive impact on soil quality, which directly affects plant productivity. Li et al. [29] and Liu et al. [30] found that long-term application of organic fertilizers considerably increased soil organic matter and nutrient levels, and SWC, collectively contributing to improve crop yield. These findings are corroborated by our results (Table 1, Figure 1). The direct addition of organic matter from organic fertilizer was probably the main cause since it contained a variety of active substances that might promote microbial activity, which is essential for the cycling and transformation of nutrients and, subsequently, improving nutrient availability [30,31]. The combined application of chemical and organic fertilizer can slow down the release and loss of nutrients, improving the efficiency of nutrient utilization while increasing crop yield [28,29,32]. Correlation analysis in our study further substantiated that variations in soil nutrients status under different fertilization strategies were the main factors influencing *C. oleifera* yields (Figure S2). Most studies have shown that the combination of organic and chemical fertilizer has the potential to alleviate soil acidification, as organic fertilizers introduce bicarbonates and carbonates that elevated soil pH, while the organic acids they contain characterized by phenolic hydroxyl and carboxyl groups buffer soil acidity [29]. Contrarily, our findings revealed a decrease in soil pH following fertilizer application, consistent with previous studies [33,34]. Fertilization may reduce the efficiency of nitrogen usage, raising nitrate and acidity levels in the soil solution through the nitrification process. To counteract the additional acidity, a quicker reduction in base cations occurs, resulting in a quicker and more significant drop in pH [35]. The above results indicate that organic fertilizer substitution can substantially improve soil nutrient status and availability, thereby promoting the yield improvement of *C. oleifera*.

### 4.2. Effect of Fertilization on Soil Bacteria and Fungi Diversity

Soil microbial diversity is widely acknowledged as a crucial indicator for assessing soil ecosystem functions [36]. It is generally accepted that the greater soil microbial diversity enhances resistance to external environmental disturbances and contributes to the stability of soil ecosystem [4,36]. Fertilization applications may affect soil microbial reproduction and growth by modifying their growth environment, thereby impacting microbial diversity [4,37]. Notably, the diversity of soil fungi and bacteria exhibits significant variation in response to multiple fertilization strategies [38]. For instance, several studies have reported that organic fertilization practices can increase the diversity of soil bacteria and fungi [39,40,41], while others have reported neutral or even detrimental effects [32,42,43]. In this study, LOM and HOM increased soil bacterial diversity, aligning with the findings of Bebber, D.P. et al. [17]. This enhancement may be attributed to organic fertilizers improving soil nutrient availability and providing a more diverse substrate for bacterial growth [44,45]. Nonetheless, soil fungi diversity indexes varied with organic fertilizer addition replacement proportion, e.g., LOM considerably increased Shannon and Simpson indexes of soil fungi while HOM notably reduced them. This observation is consistent with Wang et al. [45], who proved that excessive application of organic fertilizers in conjunction with chemical fertilizers can reduce soil fungal diversity. Thus, an appropriate fertilization strategy is more conducive to the construction of soil microbial diversity.

In addition, PCoA and PERMANOVA results also revealed that soil bacterial and fungal communities changed differently depending on fertilization treatments fertilization treatments, which is consistent with the reports by Morugán-Coronado et al. [46]. These analyses further revealed that soil bacterial communities exhibited greater sensitivity to the substitution of organic fertilizer compared to fungal communities, supporting the views of previous authors [47]. Such results also demonstrated that adding exogenous substances to the soil can disrupt the equilibrium of microbial species by altering soil properties, thereby inhibiting certain species while promoting the proliferation of others [48,49]. Ai et al. [38] and Pahalvi, H.N. [14] also found that soil fungi tend to be more resilient to environment change than soil bacterial communities.

### 4.3. Effect of Fertilization on Soil Fungi and Bacteria Community and Structure

Soil microbial communities are involved in a variety of ecosystem processes, given their complexity and opacity. The main approach to understanding soil microbial communities is to categorize them into ecologically essential groups [31,49]. Microorganisms are typically classified into two main catabolic groups: bacteria and fungi. In our study, HOM increased the abundance of soil fungi, consistent with findings by Li et al. [50], who demonstrated that soil fungi reproduction was enriched with organic fertilizer added. Thus, this increment may have a decisive impact on the balanced distribution of soil fungi and bacteria. The bacteria:fungi (B:F) ratio is frequently regarded as an indicator to display the balance between fungal and bacterial energy channels [51]. Soil fungi are generally associated with low nutrient availability and slower organic matter decomposition rates, while bacteria thrive in soils with higher nutrient availability and faster decomposition rates [52]. The B:F ratio also reflects soil nutrient status, and an increase in this ratio may imply reduced nutrient availability and slower soil microbe growth rates [19]. Based on this, the B:F ratio is expected to be higher in the CK and lower in HOM soil. In fact, our findings partially support this hypothesis, as we observed a lower ratio of B:F in HOM and a higher ratio in NPK (Figure 3b). These results can be attributed to the distinct variation in the abundance of soil fungi and bacteria between the NPK and HOM treatments compared to CK (Figure 3a).

The application of organic fertilizer resulted in a notable change in the composition of fungal and bacterial communities. In this study, the dominant bacterial phyla (>10% relative abundance), such as *Acidobacteriota* and *Chloroflexota*, are typically characterized as oligotrophic groups commonly found in nutrient-deficient habitats [53]. Notably, HOM soil had a lower abundance of these two phyla, suggesting an enhancement in soil nutrient status. This observation aligns with the significant increase in soil fertility indicators, including SOC, TN, TP and AN contents, under the HOM treatment, which likely suppressed the proliferation of oligotrophic communities. Subsequent step-wise correlation relationship analysis further confirmed that SOC and TN exerted significant impact on the abundance of *Acidobacteriota* and *Chloroflexota* (Table S2). Conversely, our study found that the copiotrophic phylum, i.e., *Pseudomonas,* was prevalent in HOM soils (average abundance > 29%). Tao et al. [54] also reported that higher organic fertilizer addition may stimulate the growth of indigenous *Pseudomonas* phylum. To elucidate the effects of different fertilization strategies on bacterial phyla at a finer taxonomic resolution, LDA was carried out to identify species-level biomarkers. Our findings revealed that most biomarkers in HOM treatment belonged to the phylum *Pseudomonadota* phylum, suggesting that species within *Pseudomonadota* are key contributors to the differences in soil microbiology observed between the HOM and other treatments (Figure 6a). It is generally believed that many genera of *Pseudomonas* are closely linked to plant disease resistance [55]. Our research shown that approximately half of the biomarkers identified under HOM treatments belonged to the genus *Rhodanobacter*, known for its strong tolerance to environments stress [56,57], and its antagonistic activity against the root-rot fungal pathogen *Fusarium* solani, as well as its role in promoting plant growth [58]. Thus, HOM treatment may have increased soil resistance to pathogenic bacteria.

In terms of soil fungi, the phyla *Mucoromycota, Ascomycota* and *Basidiomycota* were identified as the predominant fungal groups in soil. These three phyla constituted an average of 96.6% of the total fungal sequences, which is similar to the observation made by Mannaa et al. [58]. Among that, the relative abundance of *Mucoromycota* was considerably higher in HOM than in NPK treatment, indicating higher reduction ratio of chemical fertilizer combined with organic fertilizer substitution may preferentially promote the growth of *Mucoromycota*. In our research, *Mucoromycota* species, especially *Arbuscular mycorrhizal* fungi (AMF), were regarded as key biomarkers in HOM based on LDA results (Figure 6b). The organic fertilization addition likely provided external nutrient substrates that stimulated AMF reproduction [59]. AMF perform several essential ecological functions, such as plant nutrient absorption and resistance to environmental stress [60,61], which may explain the elevated relative abundance of *Mucoromycota* observed in HOM. In agricultural ecosystem, *Fusarium* has been proven to be a pathogens fungus, which can cause catastrophic crop diseases such as *Fusarium* root rot and banana *Fusarium* wilt disease [62,63,64]. In this study, two *Fusarium* species, *F. oxysporum* and *F. euwallaceae,* were detected predominantly in NPK treatments, indicating that NPK may promote the proliferation of specific pathogenic fungi.

## 5. Conclusions

Studies have demonstrated that partial organic substitution serves as a viable strategy to enhance *C. oleifera* yield and soil fertility, with higher substitution rates delivering substantial improvements. It reveals a critical microbial mechanism: organic fertilizer substitution induces a shift in the soil microbial community from bacterial to fungal dominance, enriches copiotrophic bacteria and AMF, and enhances microbial diversity, thereby resulting in a more productive soil environment. Most importantly, we identify soil organic carbon and total nitrogen as the primary drivers in this process, with direct effects on yield and microbial community structure. Consequently, we advise implementing a high-rate organic fertilizer substitution strategy in comparable planting areas. Further research should refine the optimal substitution ration and formula in *C. oleifera* production, and elucidate its impact on ecosystem microbial function.

## Figures and Tables

**Table 1:** Soil properties under different fertilization treatments.

	CK	NPK	LOM	HOM
BD, g cm^−3^	1.31 ± 0.02 a	1.33 ± 0.05 a	1.34 ± 0.02 a	1.21 ± 0.02 b
SWC, %	14.1 ± 0.27 b	15.2 ± 0.60 ab	14.5 ± 0.60 b	16.6 ± 0.73 a
pH	4.79 ± 0.10 a	4.34 ± 0.16 b	4.17 ± 0.10 b	4.37 ± 0.05 b
SOC, g kg^−1^	2.09 ± 0.34 c	2.78 ± 0.54 bc	4.60 ± 0.46 ab	5.09 ± 0.95 a
TN, g kg^−1^	0.21 ± 0.03 b	0.28 ± 0.05 b	0.44 ± 0.03 ab	0.58 ± 0.15 a
TP, g kg^−1^	0.77 ± 0.38 b	0.37 ± 0.02 b	1.35 ± 0.92 a	128 ± 94.2 a
AN, mg kg^−1^	29.3 ± 3.94 b	48.5 ± 9.53 b	56.0 ± 9.00 b	264 ± 27.3 a
AP, mg kg^−1^	0.78 ± 0.38	0.38 ± 0.03	1.35 ± 0.92	36.0 ± 7.19
AK, mg kg^−1^	18.8 ± 0.73 a	14.9 ± 0.64 b	16.9 ± 0.66 a	16.9 ± 0.43 a
C:N	10.2 ± 0.83	9.78 ± 0.34	10.5 ± 0.44	10.5 ± 2.19
C:P	22.3 ± 3.05	33.2 ± 5.44	42.0 ± 4.34	34.0 ± 7.96
N:P	2.23 ± 0.33	3.41 ± 0.57	3.85 ± 0.31	4.00 ± 1.03

Data present means ± standard error, different letters represent significant difference among four fertilization treatments (*p* < 0.05). CK: without fertilizer, NPK: 100% chemical fertilizer, LOM: 30% organic fertilizer + 70% chemical fertilizer, HOM: 60% organic fertilizer + 40% chemical fertilizer. BD: bulk density; SWC: soil water content; AN: available nitrogen; AP: available phosphorus; AK: available potassium; SOC: soil organic carbon; TN: total nitrogen; TP: total phosphorus; C:N: the ratio of SOC to TN; C:P: the ratio of SOC to TP; N:P: the ratio of TN to TP.

**Table 2:** Alpha diversity of soil bacteria and fungi among four fertilization treatments.

		Chao1	ACE	Shannon	Simpson	Goods Coverage
Bacteria	CK	24,382 ± 407	23,862 ± 400	4.00 ± 0.22 c	0.91 ± 0.01 b	99.98%
	NPK	25,003 ± 220	24,509 ± 246	4.16 ± 0.05 bc	0.92 ± 0.00 b	99.98%
	LOM	24,517 ± 756	23,982 ± 754	4.52 ± 0.18 ab	0.95 ± 0.00 a	99.98%
	HOM	25,030 ± 597	24,514 ± 568	4.68 ± 0.19 a	0.95 ± 0.01 a	99.98%
Fungi	CK	955 ± 49.8 ab	939 ± 41.1 ab	4.24 ± 0.34 ab	0.90 ± 0.03 ab	99.98%
	NPK	826 ± 49.1 b	819 ± 41.6 b	4.27 ± 0.15 ab	0.92 ± 0.02 ab	99.98%
	LOM	916 ± 29.7 ab	898 ± 29.4 ab	4.85 ± 0.13 a	0.97 ± 0.00 a	99.98%
	HOM	1010 ± 52.6 a	1001 ± 50.5 a	3.48 ± 0.57 b	0.82 ± 0.06 b	99.98%

Data present means ± standard error, different lowercase letters represent significant difference among four fertilization treatments (*p* < 0.05). CK: without fertilizer, NPK: 100% chemical fertilizer, LOM: 30% organic fertilizer + 70% chemical fertilizer, HOM: 60% organic fertilizer + 40% chemical fertilizer.

## Data Availability

The raw data supporting the conclusions of this article will be made available by the authors on request.

## Supplementary Materials

The following supporting information can be downloaded at: https://www.mdpi.com/article/10.3390/microorganisms13112509/s1, Figure S1: The distribution of oligotrophs and copiotrophs (a) and the ratio of oligotrophs to copiotrophs community (b). Data present means ± standard error, different lowercase letters represent significant difference among four fertilization treatments (*p* < 0.05). CK: without fertilizer, NPK: 100% chemical fertilizer, LOM: 30% organic fertilizer + 70% chemical fertilizer, HOM: 60% organic fertilizer + 40% chemical fertilizer, C:O: the ratio of copiotrophic to oligotrophic community; Figure S2: Spearman correlation analysis heat maps of environmental factors and soil microbial composition and structure and plant yields. Red indicates a positive correlation and blue indicates a negative correlation (* *p* < 0.05, ** *p* < 0.01, *** *p* < 0.001). B_Shannon and B_Simpson indicates soil bacteria Shannon and Simpson. F_Shannon and F_Simpson indicates soil fungi Shannon and Simpson. C:O: the ratio of copiotrophic to oligotrophic community. SWC: soil water content; BD: bulk density; AN: available nitrogen; AP: available phosphorus; AK: available potassium; SOC: soil organic carbon; TN: total nitrogen; TP: total phosphorus; C:N: the ratio of SOC to TN; C:P: the ratio of SOC to TP; N:P: the ratio of TN to TP; Table S1: Metagenome sequencing statistics of each sample; Table S2: Bacterial and fungal species-level abundance differences (Relative Abundance >1%); Table S3: Results of stepwise multiple linear regression analyses showing the dependence of dominant bacteria contents on soil physicochemical variables.
