# Supplementary material for: Substituting Chemical by Organic Fertilizer Improves Soil Quality, Regulates the Soil Microbiota and Increases Yields in Camellia oleifera
Source: Microorganisms. 2025 Oct 31;13(11):2509. doi: 10.3390/microorganisms13112509 (PMC12654687; doi:10.3390/microorganisms13112509)
Supplement: Supplementary file 1 [file microorganisms-13-02509-s001.zip › microorganisms-3853066-supplementary.pdf]

Table S1 Metagenome sequencing statistics of each sample

Sample	RawReads	CleanReads	Contigs_Num	Mean Len	Max Len	Min Len	GC(%)	N50	N90
CK	79137084	79063044	510210	1084	175703	500	59.9%	1131	558
CK	80645284	80585914	651062	1056	670242	500	61.3%	1067	556
CK	77372408	77312134	558523	1032	250469	500	61.5%	1026	551
CK	78394420	78319236	686708	965	127255	500	60.5%	968	551
NPK	81502286	81449458	512991	1057	407607	500	60.3%	1067	553
NPK	80645332	80581416	588984	1002	122493	500	61.1%	999	550
NPK	78734676	78660804	667263	944	390623	500	61.3%	929	547
NPK	76713432	76655704	619727	988	442603	500	60.2%	985	550
LOM	81130508	81070546	591691	1042	181320	500	61.4%	1049	554
LOM	81323350	81256830	427567	1016	384439	500	61.2%	1005	549
LOM	78062750	77992362	604711	1088	549354	500	62.6%	1112	560
LOM	80006306	79939800	567080	951	213007	500	61.6%	922	546
HOM	82732794	82674784	485772	922	111536	500	60.9%	887	543
HOM	81420278	81354026	529112	1010	247959	500	60.8%	994	550
HOM	79885876	79837060	642610	1043	411235	500	61.5%	1058	557
HOM	78394302	78322098	516187	1058	417973	500	61.6%	1082	558

CK: without fertilizer, NPK: 100% chemical fertilizer, LOM: 30% organic fertilizer + 70% chemical fertilizer, HOM: 60% organic fertilizer + 40% chemical fertilizer.

Table S2 Bacterial and fungal species-level abundance differences (Relative Abundance >1%)

	phyla	species	CK	NPK	LOM	HOM
Bacteria	*Acidobacteriota*	*Acidobacteriota*	20.2 ± 2.59a	19.8 ± 1.94a	14.0 ± 2.01b	12.8 ± 2.44b
		Terriglobia	6.98 ± 1.52	7.96 ± 2.04	5.96 ± 1.34	5.46 ± 1.54
		Acidobacteriaceae	1.88 ± 0.45a	1.73 ± 0.44ab	1.29 ± 0.47ab	1.08 ± 0.34b
		Candidatus_Acidoferrum_typicu	1.84 ± 0.93	1.42 ± 0.80	1.06 ± 0.72	0.81 ± 0.83
	Actinomycetota	*Actinomycetes*	2.93 ± 0.68	2.79 ± 0.62	4.07 ± 1.13	4.21 ± 1.57
		Actinomycetota	1.58 ± 0.44	1.42 ± 0.20	1.87 ± 0.29	1.49 ± 0.49
		Solirubrobacterales	0.95 ± 0.47	0.72 ± 0.38	1.10 ± 0.60	1.11 ± 0.57
	Chloroflexota	*Chloroflexota*	15.7 ± 1.79a	12.1 ± 2.71b	12.2 ± 1.74b	10.1 ± 2.07b
		Ktedonobacteraceae	0.98 ± 0.33	0.83 ± 0.19	1.09 ± 0.47	0.91 ± 0.29
	Candidatus_Eremiobacterota	*Candidatus_Eremiobacteraeota*	3.65 ± 0.68	3.59 ± 0.84	2.73 ± 0.93	2.44 ± 0.72
		Candidatus_Eremiobacteraeota	3.65 ± 0.68	3.59 ± 0.84	2.73 ± 0.93	2.44 ± 0.72
	Candidatus_Dormibacteraeota	*Candidatus_Dormibacteraeota*	1.19 ± 0.48	0.80 ± 0.50	0.76 ± 0.42	0.63 ± 0.31
	Pseudomonadota	*Alphaproteobacteria*	4.44 ± 0.42	5.11 ± 0.91	5.80 ± 1.80	5.93 ± 2.45
		Gammaproteobacteria	1.54 ± 0.34b	2.64 ± 1.30ab	3.73 ± 1.37a	3.16 ± 1.09ab
		Hyphomicrobiales	0.91 ± 0.22	0.98 ± 0.27	1.21 ± 0.13	1.18 ± 0.61
Fungi	*Mucoromycota*	*Rhizophagus_irregularis*	30.4 ± 9.91a	27.0 ± 8.82ab	15.8 ± 3.45b	37.5 ± 12.83a
		Rhizophagus_clarus	8.00 ± 1.94ab	9.13 ± 1.79ab	5.18 ± 1.26b	11.51 ± 5.46a
		Glomus_cerebriforme	2.65 ± 0.96ab	2.67 ± 1.07ab	1.33 ± 0.32b	4.81 ± 2.94a
		Rhizophagus_sp._MUCL_43196	1.79 ± 0.37ab	2.02 ± 0.73ab	1.06 ± 0.44b	3.02 ± 1.69a
		Rhizopus_arrhizus	1.77 ± 0.66	3.80 ± 3.25	2.58 ± 0.60	2.46 ± 2.11
		Rhizoctonia_solan	0.82 ± 0.67ab	0.16 ± 0.18b	1.39 ± 1.48a	0.13 ± 0.09b
	Ascomycota	*Aspergillus_fumigatu*	1.33 ± 0.45	1.94 ± 1.26	2.04 ± 1.10	0.65 ± 0.72
		Macrophomina_phaseolin	0.67 ± 0.62ab	0.18 ± 0.10b	1.40 ± 1.44a	0.15 ± 0.13b
	Basidiomycota	*Gelatoporia_subvermispora*	0.74 ± 0.86ab	0.03 ± 0.03b	1.50 ± 1.65a	0.12 ± 0.15ab

Data present means ± standard error, different lowercase letters represent significant difference among four fertilization treatments ($p < 0.05$). CK: without fertilizer, NPK: 100% chemical fertilizer, LOM: 30% organic fertilizer + 70% chemical fertilizer, HOM: 60% organic fertilizer + 40% chemical fertilizer, C:O: the ratio of copiotrophic to oligotrophic community.

Table S3 Results of stepwise multiple linear regression analyses showing the dependence of dominant bacteria contents on soil physicochemical variables

Explanatory variable	Coefficient	Model R^2	*p* value
Bacteria			
Acidobacteriota			
TN	-0.57	0.28	0.02
Chloroflexota			
SOC	-0.72	0.48	0.01
Bacteroidota			
TN	0.53	0.23	0.04
Candidatus Eremiobacterota			
TN	-0.55	0.26	0.02
Candidatus Dormibacteraeota			
AK	0.63	0.35	0.01
Pseudomonadota			
TN	0.66	0.39	0.01
Fungi			
TN	0.61	0.36	0.01
N:P	-0.46	0.55	0.02

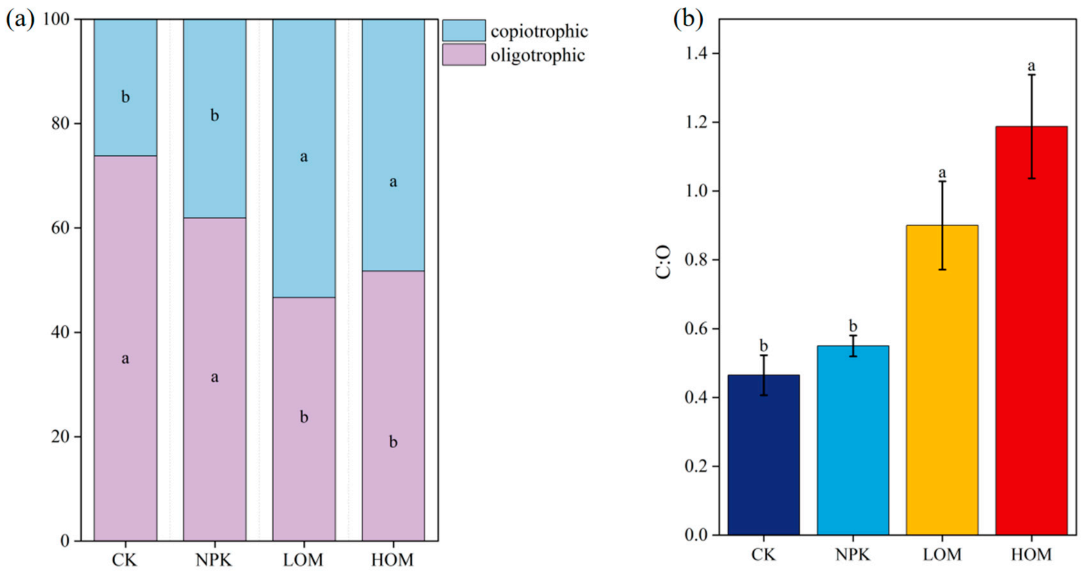

Figure S1 The distribution of oligotrophs and copiotrophs (a) and the ratio of oligotrophs to copiotrophs community (b). Data present means ± standard error, different lowcase letters represent significant difference among four fertilization treatments ($p < 0.05$). CK: without fertilizer, NPK: 100% chemical fertilizer, LOM: 30% organic fertilizer + 70% chemical fertilizer, HOM: 60% organic fertilizer + 40% chemical fertilizer, C:O: the ratio of copiotrophic to oligotrophic community.

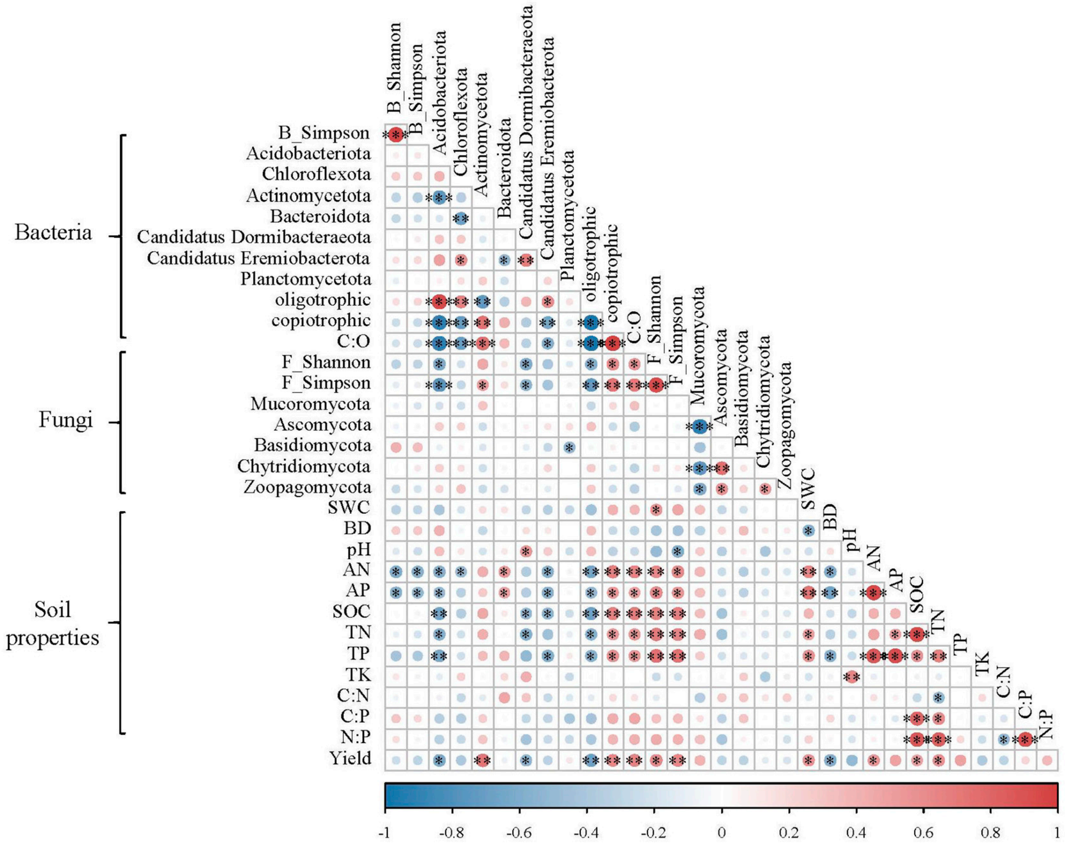

Figure S2 Spearman correlation analysis heat maps of environmental factors and soil microbial composition and structure and plant yields. Red indicates a positive correlation and blue indicates a negative correlation (* $p<0.05$, ** $p<0.01$, ***$p<0.001$). B_Shannon and B_Simpson indicates soil bacteria Shannon and Simpson. F_Shannon and F_Simpson indicates soil fungi Shannon and Simpson. C:O: the ratio of copiotrophic to oligotrophic community. SWC: soil water content; BD: bulk density; AN: available nitrogen; AP: available phosphorus; AK: available potassium; SOC: soil organic carbon; TN: total nitrogen; TP: total phosphorus; C:N: the ratio of SOC to TN; C:P: the ratio of SOC to TP; N:P: the ratio of TN to TP.
